# Analysis of Brain Structural Connectivity Networks and White Matter Integrity in Patients With Mild Cognitive Impairment

**DOI:** 10.3389/fnagi.2022.793991

**Published:** 2022-01-31

**Authors:** Maurizio Bergamino, Simona Schiavi, Alessandro Daducci, Ryan R. Walsh, Ashley M. Stokes

**Affiliations:** ^1^Barrow Neuroimaging Innovation Center, Barrow Neurological Institute, Phoenix, AZ, United States; ^2^Department of Computer Science, University of Verona, Verona, Italy; ^3^Department of Neuroscience, Rehabilitation, Ophthalmology, Genetics, Maternal and Child Health (DINOGMI), University of Genoa, Genoa, Italy; ^4^Muhammad Ali Parkinson Center, Barrow Neurological Institute, Phoenix, AZ, United States

**Keywords:** Alzheimer’s disease, mild cognitive impairment, diffusion tensor imaging (DTI), tractography, structural connectivity, white matter integrity

## Abstract

White matter integrity and structural connectivity may be altered in mild cognitive impairment (MCI), and these changes may closely reflect decline in specific cognitive domains. Multi-shell diffusion data in healthy control (HC, *n* = 31) and mild cognitive impairment (MCI, *n* = 19) cohorts were downloaded from the ADNI3 database. The data were analyzed using an advanced approach to assess both white matter microstructural integrity and structural connectivity. Compared with HC, lower intracellular compartment (IC) and higher isotropic (ISO) values were found in MCI. Additionally, significant correlations were found between IC and Montreal Cognitive Assessment (MoCA) scores in the MCI cohort. Network analysis detected structural connectivity differences between the two groups, with lower connectivity in MCI. Additionally, significant differences between HC and MCI were observed for global network efficiency. Our results demonstrate the potential of advanced diffusion MRI biomarkers for understanding brain changes in MCI.

## Introduction

Mild cognitive impairment (MCI) is defined by a mild but objective decline in cognitive function beyond that associated with normal aging; while patients with MCI do not yet meet the criteria for dementia, MCI is thought to represent a prodromal phase of Alzheimer’s disease (AD) (Metzler-Baddeley et al., 2012). More specifically, patients with MCI convert to AD at a rate of about 10–15% per year (Bruscoli and Lovestone, 2004), and thus diagnostic biomarkers for MCI and prognostic biomarkers for conversion along the spectrum of AD are critical for clinical care, disease management, and early intervention. While amyloid and tau pathology increase over time decades before cognitive changes are clinically observed, previous studies have suggested that incipient cognitive changes could be associated with white matter (WM) microstructural changes in cortical and subcortical regions (Zhuang et al., 2013; Luo et al., 2019).

Microscopic WM changes with aging and AD can be probed using advanced diffusion magnetic resonance imaging (dMRI) methods Bergamino et al., 2020, 2021b), revealing the underlying microstructural integrity and anatomical connectivity of the brain (Shim et al., 2017; Mayo et al., 2018). More specifically, microstructural integrity can be assessed by fitting a relevant model to obtain voxel-wise measures related to the local diffusion of water around axons, while tractography can yield biomarkers related to the large-scale structural connectivity of the brain. The most common model for dMRI is diffusion tensor imaging (DTI), where the resulting DTI parameters have previously demonstrated disease-related effects in WM in MCI and AD (Nir et al., 2013). However, the limitations of DTI are increasingly recognized, and more advanced acquisition and analysis models have been developed. For instance, standard DTI-derived metrics [e.g., fractional anisotropy (FA)] are influenced by contributions of different brain tissue compartments, including cerebral spinal fluid (CSF) and extracellular free water (Pierpaoli et al., 1996); we recently showed that these partial volume effects reduce the accuracy of DTI metrics in aging and AD populations (Bergamino et al., 2021b). Additionally, the standard single-tensor DTI model is based on a Gaussian diffusion assumption, which has only a single directional maximum; as a result, DTI cannot resolve multiple fiber orientations within a voxel (Tuch et al., 2002). This has led to the development of more complex, higher order models (Zhan et al., 2015) to resolve multiple intravoxel fiber orientations. One such model, the ball & stick model (Behrens et al., 2003), simultaneously accounts for extracellular free water diffusion (ball component) and models multiple fiber orientations (stick component). A previous study using this model, combined with a probabilistic tractography method, showed superior performance in differentiating between normal controls and MCI (Zhan et al., 2015).

Tractography is an advanced technique that enables reconstruction of major fiber tract pathways in the brain by mapping the trajectory of voxel-wise fiber orientations. Structural connectivity, which generates streamlines as a proxy for WM fiber bundles, can be subsequently inferred from tractography to reveal the anatomical organization of structural brain networks (Yeh et al., 2021). In the context of AD, previous studies have demonstrated that both structural and functional brain networks may be disrupted (Brier et al., 2014; Peraza et al., 2019). One challenge with tractography and structural connectivity is that these methods generate a significant number of false-positive connections between brain regions (Maier-Hein et al., 2017). In order to robustly decrease the number of false positives, Daducci et al. developed the Convex Optimization Modeling for Microstructure Informed Tractography (COMMIT) (Daducci et al., 2015) framework and later the COMMIT2 (Schiavi et al., 2020a) framework, which both aim to re-establish the link between tractography and tissue microstructure. The COMMIT framework effectively combines tractography with microstructural features of the tissue to enhance the robustness of connectivity estimates; however, this method is not effective for reducing false positives. To reduce the incidence of false positives, COMMIT2 aims to recover the connectome that best explains the diffusion-weighted signal while simultaneously minimizing the number of bundles. Through these two frameworks, the dMRI signal is modeled as a linear combination of local models associated with streamlines, where the weights are identified by solving a convex optimization problem. As demonstrated in Schiavi et al. (2020b), this filtering method may drastically improve the accuracy of resulting structural connectomes. To our knowledge, this method has not been assessed for differentiating between healthy aging and MCI.

In this study, we analyzed multi-shell dMRI data from the ADNI3 database^1^ using a novel, expanded approach to assess both white matter (WM) integrity and structural connectivity between healthy controls (HC) and a cohort with MCI. The COMMIT2 framework with ball & sticks forward model was used to remove false positive brain connections by assigning to each streamline its contribution to the diffusion MRI signal. Additionally, the isotropic (ISO) and restricted intra-cellular (IC) signal fractions maps obtained from the COMMIT2 global fitting were also used to perform voxel-wise analyses. The correlation between IC and ISO signal fractions maps and cognitive assessment scores was assessed. Lastly, we analyzed global and local network measures obtained from the connectomes using the brain connectivity toolbox (BCT). The overall purpose of this study was to assess WM microstructural differences between HC and MCI cohorts using this advanced analysis framework based on COMMIT2.

## Materials and Methods

### Subjects

Thirty-one HC [age mean ± standard deviation (S.D.) = 70 ± 6 years; 20 females] and 19 MCI (71 ± 9 years; 8 females) were included in this study. Subjects completed the Mini-Mental State Exam (MMSE) (Folstein et al., 1975) and the Montreal Cognitive Assessment (MoCA) (Nasreddine et al., 2005). The complete subject characteristics and cognitive scores are shown in Table 1. All data were downloaded from ADNI3, and only subjects with multi-shell DTI available were included in this study.

**TABLE 1:** Complete subjects and cognitive scores characteristics.

	**N (females)**	**Age (SD)**	**MMSE**	**MoCA**
HC	31 (20)	70 (6)	28.6 (2.0)	25.0 (2.3)
MCI	19 (8)	71 (9)	27.6 (2.3)	23.1 (2.5)
*Chi-Square*	χ^2^ = 2.401; *p* = 0.121	–	–	–
*Student’s test*	–	*t* = −0.894; *p* = 0.376	–	–
*Mann–Whitney U test*	–	–	*Z* = 2.108; *p* = 0.035	*Z* = 2.080; *p* = 0.038

### Data Availability

Data used in the preparation of this article were obtained from the Alzheimer’s Disease Neuroimaging Initiative (ADNI) database (adni.loni.usc.edu). The ADNI was launched in 2003 as a public-private partnership, led by Principal Investigator Michael W. Weiner, MD. The primary goal of ADNI has been to test whether serial magnetic resonance imaging (MRI), positron emission tomography (PET), other biological markers, and clinical and neuropsychological assessment can be combined to measure the progression of mild cognitive impairment (MCI) and early Alzheimer’s disease (AD). For up-to-date information, see www.adni-info.org.

### Image Acquisition

Axial diffusion MRI data were acquired at 3T using a multi-shell DTI acquisition (SIEMENS Magnetom Prisma Fit Scanner) with the following parameters: 114 diffusion-encoding directions [*b* values: 500 (6 directions), 1,000 (48 directions), and 2,000 (60 directions) s/mm^2^; TR/TE: 3,400/71 ms; flip-angle = 90°; matrix: 256 × 256; voxel size 2.0 × 2.0 mm; slice thickness: 2.0 mm; number of averages = 1] and 13 non-diffusion-weighted images (B0 images). Accelerated sagittal T1-weighted (T1-w) anatomical images were acquired using a 3D magnetization-prepared rapid acquisition gradient echo (MP-RAGE) sequence with the following acquisition parameters: repetition time / echo time (TR/TE), 2,300/2.95 ms; acquisition matrix, 208 × 208; voxel size, 1.0 × 1.0 mm; slice thickness, 1.0 mm; flip angle = 9°.

### T1-Weighted Image Processing

All MPRAGE were converted to NIFTI format using *dcm2niix*. The MPRAGE images were used for segmentation by FreeSurfer^2^, yielding the Desikan-Killiany parcellation atlas (Desikan et al., 2006) for each subject (file: *aparc+aseg.mgz*). Brain extraction on the MPRAGE images was performed by *ROBEX*^3^ (Iglesias et al., 2011). Using *5ttgen* [MRtrix3^4^, (Tournier et al., 2019)] and the MPRAGE images, we generated the five-tissue-type (5TT) segmented tissue image suitable for use in the anatomically constrained tractography (ACT).

### Diffusion Magnetic Resonance Imaging Processing

Similar to the MPRAGE, all dMRI were converted to NIFTI format using *dcm2niix* and were preprocessed using Mrtrix3, FSL^5^ (Jenkinson et al., 2012), and the Advanced Normalization Tool (ANTs)^6^. dMRI pre-processing included: (1) denoising by *dwidenoise* (MRtrix3), (2) alignment, distortion, and eddy-currents corrections by *eddy* (FSL), and (3) bias field correction (ANTs). The *eddy* QC tools were used to evaluate the quality of the dMRI dataset. Slices with signal loss caused by subject movement coinciding with the diffusion encoding were detected and replaced by predictions made by a Gaussian process. Brain extraction on the B0 images was performed by *dwi2mask* (MRtrix3). Using *dwi2response* with *msmt_5tt* algorithm (MRtrix3), we estimated the response function(s), and the *dwi2fod* with *msmt_csd* algorithm (MRtrix3) was used to estimate fiber orientation distributions. For coregistration between MPRAGE-based 5TT images and dMRI space, maps of FA were created by a weighted least square fitting procedure (*dtifit*; FSL), and the MPRAGE images were coregistered to FA using a rigid+affine algorithm through ANTs.

### Intra-Cellular and Isotropic Maps and Connectomes?

The COMMIT2 algorithm (with the following parameters: parallel diffusivity = 1.7 10^–3^ mm^2^/s and isotropic diffusivities = 1.7 10^–3^ mm^2^/s and 3.0.10^–3^ mm^2^/s) was used to create signal fractions maps of the ISO and IC compartments through the ball & stick model (Behrens et al., 2003; Panagiotaki et al., 2012). This model relates the local fiber structure to the diffusion signal by assuming different components within each voxel. The IC component can be viewed as a set of sticks where the water is restricted (purely anisotropic Gaussian motion), while the extra-cellular components (ISO) are modeled as purely (Gaussian) isotropic diffusion.

Tractography was performed [five million streamlines and the following parameters: min. length 4 mm, max. length = 200 mm, unidirectional tracking, maximum angle in degrees between successive steps = 45 (default) with backtrack option] through the probabilistic iFOD2 algorithm (Tournier et al., 2010) and the WM/gray-matter border as seed locations. The connectome was generated from the Desikan-Killiany atlas and was subsequently filtered by removing “non-connecting” streamlines. The COMMIT2 algorithm was then used to remove false positive brain connections.

All brain extracted B0 images were used to create a group-wise template using ANTs. In order to run voxel-based analysis, the IC and ISO signal fractions maps were non-linearly coregistered to the group-wise template using the coregistration matrices created by ANTs. Therefore, this template was used as the “standard” space for all analyses. All maps, in template space, were smoothed using FSL with an isotropic Gaussian kernel (sigma, 3 mm).

### Network Measures

Network measures (binarized for global efficiency and for mean strength) were computed for each subject using the Brain Connectivity Toolbox (BCT) (Rubinov and Sporns, 2010) in MATLAB (MathWorks, Natick, MA, United States). For global networks, modularity (which is a statistic that quantifies the degree to which the network may be subdivided into clearly delineated groups) and the global efficiency (which corresponds to the average inverse of the shortest path length in the network) were analyzed. For local networks, clustering coefficient (which is the degree to which nodes tend to cluster together) and mean strength (where each nodal strength corresponds to the sum of the weights of links connected to it) were evaluated.

### Statistical Analysis

Age, MMSE, and MoCA scores are presented as mean and S.D. for each group. Differences in age were evaluated by the Student’s *t*-test, while differences in sex were evaluated by the $\chi^{2}$ test. Differences in cognitive test scores were assessed using the Mann-Whitney *U* test. Table 1 shows the patient information and statistical analysis.

The analysis of covariance model (ANCOVA), with age and gender as covariates, was used to evaluate differences between MCI and HC for the IC and ISO signal fractions maps at the voxel-based level using *randomise* (FSL). Structural connectivity was analyzed by *connectomestats* (Mrtrix3), which runs the group-wise statistics at the edge level by using non-parametric permutation testing. The network-based statistic (NBS) (Zalesky et al., 2010) algorithm was performed with three different threshold values for t: *t*_thr_ = 2.5, *t*_thr_ = 3.0, and *t*_thr_ = 3.5. For both analyses, significance was determined using a non-parametric test with 5,000 permutations; the family-wise error (FWE) rate was controlled at the 0.05 level for multiple comparisons.

Using an *in-house* R script^7^, ANCOVA was performed for the Hedges’ *g* effect size analysis (for both IC and ISO maps and structural connectivity), which in this study was set at *g* > 0.50 for medium effect and *g* > 0.82 for large effect (α= 0.05; power = 80%; two groups, *n*_1_ = 19 and *n*_2_ = 31).

Voxel-based correlations between cognitive assessment scores and IC and ISO signal fractions maps in the MCI cohort were assessed through an in-house MATLAB script with Spearman’s correlations at *p* < 0.05 significance (FDR-corrected). Differences between network measures were assessed using the two-sample Student’s *t*-test.

White matter clusters (shown in MNI-152 1-mm space) were labeled using the Montreal Neurological Institute and Hospital (MNI) structural (Hua et al., 2008) and the Harvard-Oxford sub-cortical structural atlases (Desikan et al., 2006).

## Results

### Demographic Results

The MMSE score was available for all participants, while the MoCA score was available for 28 (of 31) HC and 15 (of 19) MCI subjects. The two groups did not differ significantly in age (*t*-test: *t* = −0.894, *p* = 0.376) or sex ($\chi^{2}$ = 2.401, *p* = 0.121) but did differ in MMSE and MoCA (Mann-Whitney *U* test: *Z* = 2.108, *p* = 0.035; *Z* = 2.080, *p* = 0.038, respectively). Demographic results are shown in Table 1.

### Voxel-Based Analysis of Intra-Cellular and Isotropic Maps

**FIGURE 1:**
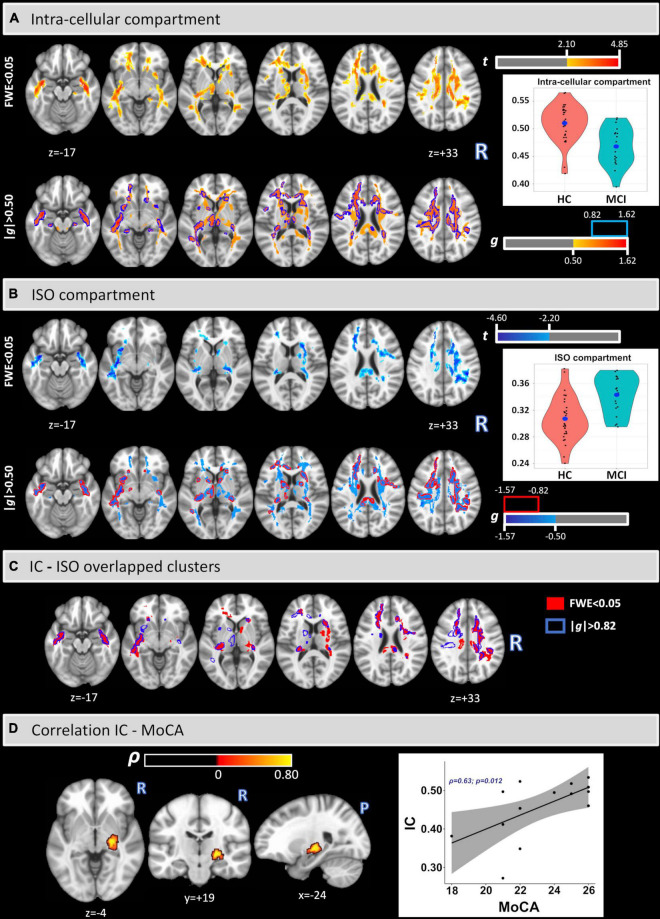
Voxel-based analysis of IC and ISO signal fraction maps **(A,B)**. Lower IC and higher ISO values were found in MCI in several white matter regions and tracts. The clusters and the coordinates are in MNI152 space. Overlapped clusters by FWE < 0.05 and | g| > 0.82 **(C)**. Significant voxel-wise Spearman’s correlations were found between IC and MoCA scores in the MCI cohort **(D)**.

Figure 1, panels (A,B), show the voxel-based analysis for the IC and ISO metrics between HC and MCI groups by using an FWE < 0.05 (top) and an effect-size *g* > 0.50 (medium effect) with border clusters at *g* > 0.82 (large effect) (bottom). Compared with HC, lower IC values were found in MCI in much of the WM, while higher ISO values were found in MCI principally in the uncinated fasciculus (UF), corpus callosum (CC), retrolenticular part of internal capsule, corona radiata (CR), sagittal stratum (SS), and the external capsule (EC). It is important to note that the results were confirmed by both FWE < 0.05 and large effect-size. The corresponding significant cluster locations, for both analyses, are reported as percent volume, t, and *g* (for large effect) values in Table 2.

**TABLE 2:** Significant clusters for IC and ISO with FWE < 0.05 and | *g*| > 0.82.

	IC				ISO			
	FWE < 0.05		| *g*| > 0.82		FWE < 0.05		| *g*| > 0.82	
JHU white-matter tractography	% vol	*t*	% vol	*g*	% vol	*t*	% vol	*g*
Anterior thalamic radiation	18.01	2.612	10.18	0.988	5.14	−2.708	7.12	−0.969
Cortical spinal tract	6.89	2.516	5.15	0.932	4.93	−2.538	7.07	−0.916
Cingulum cingulate gyrus	16.44	2.660	6.86	1.002	5.85	−2.564	5.62	−0.939
Cingulum Hippo L	–	–	6.19	1.039	–	–	–	–
Cingulum Hippo R	14.59	2.784	6.41	1.013	–	–	–	–
Forceps Minor	12.46	2.626	6.57	1.015	4.02	−2.698	3.44	−1.020
Inferior fronto-occipital fasc	18.18	2.73	8.64	1.031	6.80	−2.89	5.52	−1.01
Inferior Longitudinal fasc	15.16	2.830	7.18	1.010	5.81	−2.92	5.62	−0.95
Superior Longitudinal fasc	7.18	2.719	2.19	0.969	4.52	−2.840	4.50	−0.942
Uncinate fasc	28.09	2.822	13.31	1.073	12.41	−3.066	10.01	−1.029

**ICBM-DTI 81**	**% vol**	***t***	**% vol**	***g***	**% vol**	***t***	**% vol**	***g***

Genu of corpus callosum	16.98	2.499	9.58	0.998	4.07	−2.557	1.36	−0.919
Body of corpus callosum	31.01	2.693	15.08	0.981	12.35	−2.601	8.00	−0.906
Splenium of corpus callosum	4.83	2.613	1.61	0.958	4.60	−2.528	3.14	−0.899
Anterior limb of internal capsule R	26.45	2.527	3.03	0.970	18.18	−2.550	10.47	−0.885
Anterior limb of internal capsule L	16.91	2.399	12.24	0.926	–	–	15.45	−0.932
Posterior limb of internal capsule R	6.64	2.297	–	–	8.05	−2.397	–	–
Posterior limb of internal capsule L	5.79	2.342	1.07	0.890	0.43	−2.554	3.00	−0.894
Retrolenticular part of internal capsule R	42.74	2.431	–	–	34.02	−2.646	–	–
Retrolenticular part of internal capsule L	46.96	2.689	48.18	1.063	31.58	−2.764	39.68	−1.039
Anterior corona radiata	34.14	2.579	19.03	0.978	14.47	−2.655	9.98	−0.956
Superior corona radiata	27.72	2.535	21.49	0.959	22.62	−2.676	24.43	−0.915
Posterior corona radiata R	29.40	2.711	21.38	0.969	23.61	−2.560	14.70	−0.924
Posterior corona radiata L	20.95	2.465	25.70	0.935	–	–	3.02	−0.893
Posterior thalamic radiation R	16.22	2.603	–	–	2.10	−2.301	–	–
Posterior thalamic radiation L	21.50	2.523	14.59	0.918	–	–	–	–
Sagittal stratum	40.24	2.835	21.94	1.077	20.31	−3.022	19.42	−0.952
External capsule	18.29	3.053	8.28	1.058	15.08	−3.073	12.04	−1.007
Superior fronto-occipital fasciculus	60.02	2.430	31.25	0.929	8.09	−2.407	9.70	−0.879
Tapetum R	2.25	2.347	–	–	–	–	–	–

*“% vol” column shows the percent of the volume covered of the clusters in the corresponding atlas area.*

Figure 1 panel (C) shows the overlapped clusters between IC and ISO as calculated with an FWE < 0.05 (clusters in red) and an effect-size *g* > 0.82 (borders in blue). Additionally, Table 3 provides the relative volumes, t, and *g* values for all overlapped clusters.

**TABLE 3:** Significant overlapped clusters for both FWE < 0.05 and | *g*| > 0.82 for IC and ISO.

	FWE clusters			Effect-size clusters		
JHU white-matter tractography	% vol	t (IC)	t (ISO)	% vol	*g* (IC)	*g* (ISO)
Anterior Thalamic Radiation	4.97	2.848	−2.715	4.63	1.031	−0.999
Cortical spinal tract L	–	–	–	3.93	0.931	−0.933
Cortical spinal tract R	7.07	2.621	−2.624	4.36	0.942	−0.912
Cingulum cingulate gyrus	4.79	2.671	−2.589	3.69	1.023	−0.973
Forceps Minor	3.71	2.860	−2.706	2.92	1.090	−1.027
Inferior fronto-occipital fasc	5.63	3.096	−2.883	3.94	1.103	−1.041
Inferior Longitudinal fasc	5.38	3.29	−2.93	4.19	1.05	−0.97
Superior Longitudinal fasc	2.85	2.82	−2.93	1.52	0.98	−0.94
Uncinate fasc	9.88	3.33	−3.02	7.58	1.11	−1.05

**ICBM-DTI 81**	**% vol**	***t* (IC)**	***t* (ISO)**	**% vol**	***g* (IC)**	***g* (ISO)**

Genu of corpus callosum	3.16	2.522	−2.569	1.36	1.093	−0.919
Body of corpus callosum	10.60	2.837	−2.615	5.01	0.992	−0.916
Splenium of corpus callosum	3.07	2.736	−2.562	1.15	0.968	−0.891
Anterior limb of internal capsule R	18.18	2.618	−2.550	2.75	0.975	−0.906
Anterior limb of internal capsule L	–	–	–	9.62	0.931	−0.955
Posterior limb of internal capsule R	6.04	2.306	−2.430	–	–	–
Posterior limb of internal capsule L	0.43	2.439	−2.554	0.64	0.887	−0.973
Retrolenticular part of internal capsule R	28.22	2.514	−2.673	–	–	–
Retrolenticular part of internal capsule L	30.77	2.870	−2.773	37.25	1.100	−1.049
Anterior corona radiata	13.18	2.714	−2.684	9.12	1.014	−0.964
Superior corona radiata	17.06	2.621	−2.772	17.19	0.968	−0.925
Posterior corona radiata R	18.93	2.860	−2.599	10.91	0.995	−0.935
Posterior thalamic radiation R	1.91	2.438	−2.309	–	–	–
Sagittal stratum	15.74	3.39	−3.04	11.32	1.150	−0.991
External capsule	9.37	3.245	−3.206	7.09	1.07	−1.06
Superior fronto-occipital fasciculus R	10.42	2.421	−2.303	–	–	–
Superior fronto-occipital fasciculus L	5.77	2.970	−2.511	15.38	0.977	−0.906

*“% vol” column shows the percent of the volume covered of the clusters in the corresponding atlas area.*

Figure 1 panel (D) shows the voxel-based Spearman’s correlation between IC and MoCA. A significant cluster was found covering part of the right cortical spinal tract (CST) (% volume = 1.5%; ρ = 0.74), right inferior longitudinal fasciculus (ILF) (0.13%; ρ = 0.74), right cerebral peduncle (CP) (3.90%; ρ = 0.73), right posterior limb of the internal capsule (3.62%; ρ = 0.74), and the right retrolenticular part of the internal capsule (3.32%; ρ = 0.73). No significant correlations were found between IC and MMSE or for the ISO metric.

### Structural Connectivity

**FIGURE 2:**
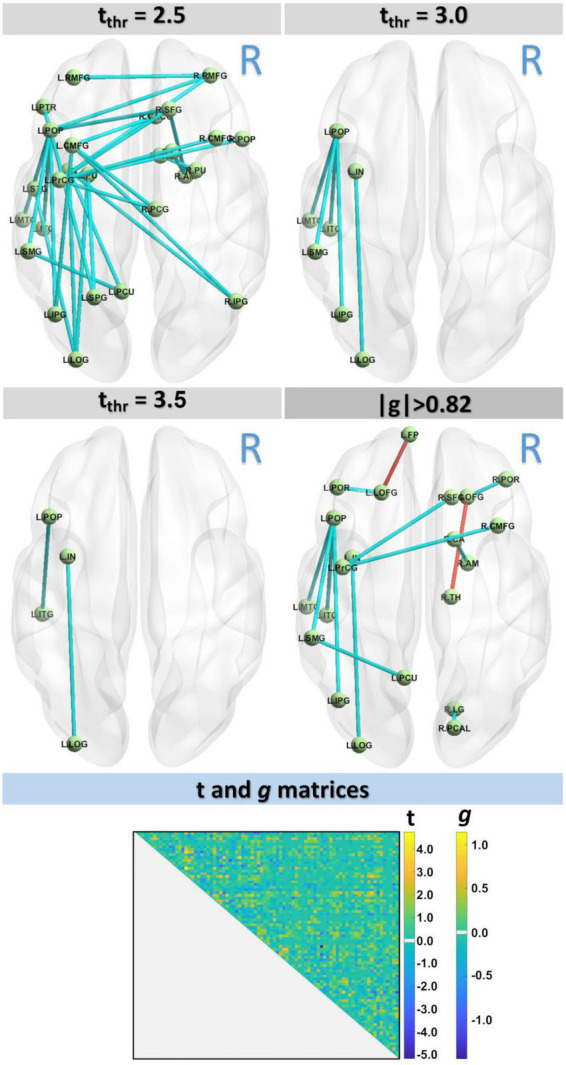
Connectivity analysis with three *t*-values thresholds, *t*_thr_ = 2.5, *t*_thr_ = 3.0, and *t*_thr_ = 3.5, showed lower connectivity in MCI group. Results were also confirmed by the effect-size analysis.

Figure 2 shows the differences in structural connectivity (using the NBS algorithm) at FWE < 0.05 between HC and MCI cohorts by three different *t*-value thresholds: *t*_thr_ = 2.5, *t*_thr_ = 3.0, and *t*_thr_ = 3.5. Additionally, the structural connectivity differences for large effect size are also shown. Compared with the HC group, lower connectivity in the MCI group was observed across all three thresholds. With increasing t_thr_, fewer connectivity differences were observed, with left hemispheric differences persisting at higher t_thr_. At the highest threshold tested (*t*_thr_ = 3.5), lower structural connectivity was found in MCI between the nodes L.POP (ctx-lh-parsopercularis) and L.ITG (ctx-lh-inferiortemporal) (*t* = 3.883, *g* = 1.114) and between the nodes L.IN (ctx-lh-insula) L.LOG (ctx-lh-lateraloccipital) (*t* = 3.997, *g* = 1.146). These results are also confirmed by the effect-size analysis, where these two edges had highest values of *g*. Although FWE analysis did not find higher connectivity in MCI, higher connectivity was observed from effect-size analysis in MCI between nodes R.TH (Right-Thalamus) and R.LOFG (ctx-rh-lateralorbitofrontal) (*t* = −3.033, *g* = −0.925) and between nodes L.LOFG (ctx-lh-lateralorbitofrontal) and L.FP (ctx-lh-frontalpole) (*t* = −3.209, *g* = −0.960). Table 4 shows the complete connectometry results.

**TABLE 4:** Connectivity results with NBS (Figure 2) by different t thresholds: *t*_thr_ = 2.5, *t*_thr_ = 3.0, *t*_thr_ = 3.5, and by the effect-size *g* > 0.82.

HC > MCI								
node 1		node 2		*t*	(*t*_thr_ = 2.5)	(*t*_thr_ = 3.0)	(*t*_thr_ = 3.5)	effect-size (*g* > 0.82)
R.RMFG	ctx-rh-rostralmiddlefrontal	L.POP	ctx-lh-parsopercularis	2.711	☑			
L.SPG	ctx-lh-superiorparietal	L.POP	ctx-lh-parsopercularis	2.716	☑			
R.PCG	ctx-rh-posteriorcingulate	L.CMFG	ctx-lh-caudalmiddlefrontal	2.729	☑			
R.AC	Right-Accumbens-area	R.PU	Right-Putamen	2.734	☑			
L.IPG	ctx-lh-inferiorparietal	L.CMFG	ctx-lh-caudalmiddlefrontal	2.736	☑			
R.CACG	ctx-rh-caudalanteriorcingulate	L.CMFG	ctx-lh-caudalmiddlefrontal	2.765	☑			
R.RMFG	ctx-rh-rostralmiddlefrontal	L.RMFG	ctx-lh-rostralmiddlefrontal	2.784	☑			
R.RMFG	ctx-rh-rostralmiddlefrontal	L.PrCG	ctx-lh-precentral	2.803	☑			
L.IN	ctx-lh-insula	L.PCU	ctx-lh-precuneus	2.808	☑			
L.PU	Left-Putamen	L.SPG	ctx-lh-superiorparietal	2.883	☑			
L.PTR	ctx-lh-parstriangularis	L.POP	ctx-lh-parsopercularis	2.929	☑			
R.POP	ctx-rh-parsopercularis	L.PrCG	ctx-lh-precentral	2.930	☑			
R.AC	Right-Accumbens-area	R.CA	Right-Caudate	2.941	☑			
R.PCG	ctx-rh-posteriorcingulate	L.PrCG	ctx-lh-precentral	2.946	☑			
L.STG	ctx-lh-superiortemporal	L.LOG	ctx-lh-lateraloccipital	2.959	☑			
R.IPG	ctx-rh-inferiorparietal	L.IN	ctx-lh-insula	2.960	☑			
L.PU	Left-Putamen	L.LOG	ctx-lh-lateraloccipital	2.971	☑			
R.SFG	ctx-rh-superiorfrontal	L.POP	ctx-lh-parsopercularis	2.995	☑			
R.SFG	ctx-rh-superiorfrontal	R.AM	Right-Amygdala	3.037	☑			
R.IPG	ctx-rh-inferiorparietal	L.CMFG	ctx-lh-caudalmiddlefrontal	3.063	☑			
R.CMFG	ctx-rh-caudalmiddlefrontal	L.PrCG	ctx-lh-precentral	3.139	☑			☑ (*g* = 0.870)
L.SMG	ctx-lh-supramarginal	L.PCU	ctx-lh-precuneus	3.145	☑			☑ (*g* = 0.875)
L.LOFG	ctx-lh-lateralorbitofrontal	L.POR	ctx-lh-parsorbitalis	3.184				☑ (*g* = 0.913)
R.LG	ctx-rh-lingual	R.PCAL	ctx-rh-pericalcarine	3.189				☑ (*g* = 0.915)
L.SMG	ctx-lh-supramarginal	L.POP	ctx-lh-parsopercularis	3.294	☑	☑		☑ (*g* = 0.945)
R.LOFG	ctx-rh-lateralorbitofrontal	R.POR	ctx-rh-parsorbitalis	3.426				☑ (*g* = 0.982)
L.POP	ctx-lh-parsopercularis	L.IPG	ctx-lh-inferiorparietal	3.561	☑	☑		☑ (*g* = 1.021)
R.AM	Right-Amygdala	R.CA	Right-Caudate	3.659	☑			☑ (*g* = 1.049)
L.POP	ctx-lh-parsopercularis	L.MTG	ctx-lh-middletemporal	3.717	☑	☑		☑ (*g* = 1.066)
R.SFG	ctx-rh-superiorfrontal	L.PrCG	ctx-lh-precentral	3.734	☑			☑ (*g* = 1.071)
L.POP	ctx-lh-parsopercularis	L.ITG	ctx-lh-inferiortemporal	3.883	☑	☑	☑	☑ (*g* = 1.114)
L.IN	ctx-lh-insula	L.LOG	ctx-lh-lateraloccipital	3.997	☑	☑	☑	☑ (*g* = 1.146)
**HC < MCI**								
**node 1**		**node 2**		**(*t*_thr_ = 2.5)**	**(*t*_thr_ = 3.0)**	**(*t*_thr_ = 3.5)**	**(*t*_thr_ = 2.5)**	**effect-size (*g* > 0.82)**
R.TH	Right-Thalamus	R.LOFG	ctx-rh-lateralorbitofrontal	−3.033				☑ (*g* = −0.920)
L.LOFG	ctx-lh-lateralorbitofrontal	L.FP	ctx-lh-frontalpole	−3.209				☑ (*g* = −0.960)

### Network Measures

**FIGURE 3:**
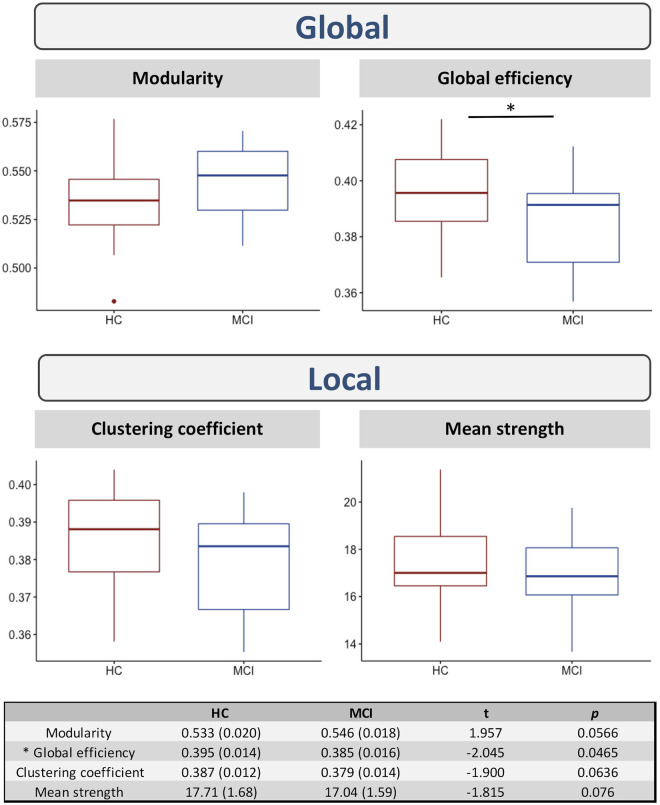
Differences in network measures between HC and MCI. ^*^ indicates significant differences between the two groups at *p(uncorrected)* < 0.05.

Figure 3 shows the differences in the network measures between the two groups. Significant differences were found in the global efficiency (*t* = −2.045; *p(uncorrected)* = 0.047). No significant differences were found in modularity, clustering coefficient, or mean strength (*p* > 0.05).

## Discussion

In this study, the ball & stick dMRI model was used to assess differences in the restricted and isotropic compartments between HC and MCI. In regions consistent with AD, the restricted compartment associated with diffusion along WM tracts was reduced in MCI, while the isotropic component was increased. The former was also found to significantly correlate with cognitive test scores, while no correlations were observed with the latter compartment. Using probabilistic tractography with COMMIT2 filtering, lower structural connectivity was also observed in the MCI cohort, supporting the concept of AD-associated disconnection. Additional evidence of connectivity changes was quantified using network-based measures, which showed global efficiency changes in MCI. Overall, these findings are consistent with WM microstructural changes in MCI.

The neuropathological hallmarks of Alzheimer’s disease are the progressive accumulation of beta-amyloid plaques and intraneuronal tau tangles, which generally follow a well-characterized spatiotemporal pattern. These changes are thought to occur as many as decades before cognitive changes and are eventually followed by neurodegeneration. However, it is increasingly recognized that neurodegenerative patterns are highly heterogenous across AD sub-groups (Dong et al., 2017; Ekman et al., 2018). Unfortunately, identification of gross tissue atrophy patterns is unlikely to modify current therapeutic targets, as early identification and thus treatment is likely a key factor in prevention. On the other hand, the neurodegenerative pathways underlying tissue atrophy are likely associated with even earlier microscopic tissue changes that are invisible to standard MRI methods but may be probed by microstructural biomarkers with dMRI. These changes in WM microstructure can include partial loss of axons, myelin, and oligodendrocytes (Sjöbeck et al., 2005).

MRI-based dMRI measures may be sensitive to early changes associated with MCI. Standard DTI-derived measures consistently show alterations associated in MCI relative to healthy controls, including decreased FA, as well as increased axial, radial, and mean diffusivities [as reviewed in Nir et al. (2013)]. WM regions implicated in DTI studies broadly include the temporal lobe and CC. Unfortunately, standard DTI metrics are known to be affected by sub-voxel neurodegeneration, which may reduce their sensitivity and accuracy to detect changes in MCI and AD (Bergamino et al., 2021b). Another drawback to standard DTI is an inability to resolve crossing fiber tracts, which reduces the specificity of DTI metrics (such as FA, AxD, RD, and MD) in regions with multiple fiber tracts. To overcome these limitations, many advanced dMRI analysis frameworks have been developed (Afzali et al., 2021). Free-water (FW-) DTI (Pasternak et al., 2009) includes an isotropic motion term in the model and may improve DTI accuracy in aging populations (Bergamino et al., 2021b). Other advanced microstructural models include the ball-&-sticks model (used herein) and neurite orientation dispersion and density imaging (NODDI) (Zhang et al., 2012). In the context of MCI, NODDI-derived metrics may improve diagnostic performance over standard DTI metrics (Fu et al., 2020). Moving beyond voxel-based methods, WM fiber orientation distributions (FODs) can be generated from constrained spherical deconvolution methods, which then enables quantification of fiber-specific metrics and improved specificity in regions with crossing fiber tracts. Reduced fiber density has been observed in both MCI (Mito et al., 2018) and AD (Luo et al., 2021) using fixel-based analysis. The COMMIT2 framework augments probabilistic FOD-based tractography using anatomically and microstructure-informed filtering; this robust analysis framework has been demonstrated to dramatically improve the specificity of the estimated brain networks without affecting their sensitivity (Schiavi et al., 2020b). In this study, the COMMIT2 framework was applied to multi-shell dMRI data from ADNI3, yielding both voxel-wise estimates of IC and ISO and the filtered tractogram.

Biophysically, the IC component represents the fraction of dMRI signal associated with WM axons. In this study, we found reduced IC in MCI relative to HC in several WM locations, such as the retrolenticular part of the internal capsule [FWE: cluster covered 43% (right) and 47% (left); effect-size: 48% (left)], UF (FWE: 28%; effect-size: 13%), ATR (FWE: 18%; effect-size: 10%), CC (FWE: 18%; effect-size: 7%), IFOF (FWE: 15%; effect-size: 7%), forceps minor (FWE: 12.5%; effect-size: 4%), inferior and superior longitudinal fasciculus (FWE: 15% and 7%, respectively; effect-size: 6 and 4.5%, respectively), and anterior/superior/posterior corona radiata (FWE: 29%; effect-size: 22%). Notably, several of these are long-range WM pathways responsible for connecting the frontal lobe with the occipital, parietal, and temporal lobes (such as the longitudinal fasciculus and IFOF), as well as ascending and descending projection fibers (such as the corona radiata and cortical spinal tract). Several of these tracts have been implicated in cognitive impairment (Dou et al., 2020) and conversion to AD (Fu et al., 2014; Dou et al., 2020). The CC is the largest WM tract in the human brain with more than 300 million fibers interconnecting the two cerebral hemispheres. Altered WM microstructure has been observed in the genu of the CC in several studies using dMRI (Lao et al., 2017; Raghavan et al., 2020), while both cross-sectional and longitudinal studies have reported atrophy of the CC in MCI and AD (Di Paola et al., 2010; Bachman et al., 2014). Relatedly, the forceps minor connects the lateral and medial frontal cortex from the genu of the CC and has been implicated in worsening cognitive decline (Luo et al., 2019). We also found a significant cluster of correlations between IC and MoCA scores that covered the right part of the CST, ILF, CP, and the retrolenticular part and posterior limb of the internal capsule. Thus, our results similarly suggest that changes in these WM regions may be biomarkers of cognitive decline in MCI. The ISO signal fraction reflects the portion of the dMRI signal explained by tissue with isotropic diffusion, and increased ISO may be indicative of sub-voxel neurodegeneration. Consistent with this interpretation, higher ISO was found in MCI in the UF (FWE: 12.5%, effect-size: 10%), CC (FWE: 7%, effect-size: 4%), retrolenticular part and right anterior limb of the internal capsule (FWE: 33 and 18%, effect-size = 39 and 10%, respectively), and the anterior/superior/posterior corona radiata (FWE: 20%, effect-size: 13%). Similar increases in the isotropic WM diffusion have been widely documented in MCI and AD using a complementary FW-DTI approach (Dumont et al., 2019; Bergamino et al., 2021b), where the FW index was significantly elevated inside the corpus callosum and fornix, indicative of AD-associated neurodegeneration. In this study, we did not find any significant correlations between the ISO metric and cognitive scores, while IC and cognitive score correlations were observed only in the right hemisphere. In contrast, group differences were essential bilateral, with no obvious trends toward laterality.

In this study, we found differences in connectivity across various thresholds and at a large effect-size. Notably, at the lowest threshold tested, connectivity changes were observed bilaterally and interhemispherically; with higher thresholds, connectivity differences persisted only in the left hemisphere. Asymmetric connectivity changes have been previously reported in MCI and AD (Yang et al., 2017), where left hemispheric changes were dominant. At a moderate threshold, lower connectivity in MCI than HC was observed between the L.POP and the L.MTG, L.ITG, L.SMG, and L.IPG nodes, as well as between the L.IN node and L.LOG node. These connectivity differences were also associated with large effect size. The nodes L.POP and L.ITG are connected by the left SLF, which connects the occipital, parietal, and temporal lobes with the frontal cortex. WM changes in the SLF have been previously reported in AD relative to healthy controls using standard DTI metrics (Meng et al., 2012). Significant structural connectivity differences in MCI and AD have also recently been associated with the SLF (Yang et al., 2021), which is a key component of the frontoparietal network. This is further consistent with studies showing decreased functional connectivity in the frontoparietal network, concomitant with altered connectivity in the default mode network, in both AD (Zhao et al., 2019) and older adults with subtle cognitive impairment (Zanchi et al., 2017). It is important to note that the SLF plays an important role in language (Madhavan et al., 2014), attention (Mesulam, 1981), and memory (Zheng et al., 2021). While no connectivity increases (for MCI) were observed across *t*-value thresholds, effect-size analysis identified two pairs of nodes with higher connectivity in MCI (L.LOFG—L.FP and R.LOFG—R.TH). Increased connectivity has been reported previously in MCI and AD cohorts (Molinuevo et al., 2014); these unexpected findings have been attributed to methodological inaccuracies or a compensatory mechanism that manifests as remodeled neural networks. As these increases in connectivity were only present in the effect-size analysis, these findings should be considered in larger studies in the future.

In addition to structural connectivity changes, we also observed differences in network measures, notably in global efficiency, using a graph theory approach to derive properties of the global brain “connectome” (Rubinov and Sporns, 2010). Global efficiency is inversely related to the path length between nodes and is typically interpreted as a measure of the system capacity for parallel transfer and integrated processing of information (Bullmore and Sporns, 2012). Decreases in global efficiency and mean clustering coefficient have previously been observed in MCI (Reijmer et al., 2013; Berlot et al., 2016); consistent with that study, we found lower global efficiency and clustering coefficient, though the latter was not significant. Global network connectivity changes have been implicated in reduced cognitive control in MCI (Berlot et al., 2016), though specific cognitive domains such as episodic memory may be less sensitive to these global network changes (and more sensitive to local connections). These findings suggest that both global and local network changes play a role in the onset of MCI, although the mechanisms underlying these changes may differ in both their pathophysiological basis and associated symptoms (Reijmer et al., 2013; Berlot et al., 2016).

There are several limitations to this study. One limitation is the low number of the subjects in the MCI group (*n* = 19). The study population was selected from ADNI3 participants that were scanned on Siemens scanners with multi-shell dMRI and 114 diffusion-encoding directions. Other ADNI protocols involve different scanner manufacturers (GE) or fewer directions and shells (54 and 30 directions only *b* = 1,000 s/mm^2^). Unfortunately, one drawback of multicenter studies is potential bias resulting from hardware and software differences between MRI scanners and acquisition parameters. We previously showed that these variances across scanners may reduce the reliability of the MR measures or even conceal the significance of the effect of interest (Bergamino et al., 2021a). For this reason, a single acquisition was selected for this study, though future studies should confirm these findings in a larger cohort. Relatedly, we provide results related to both statistical significance and effect size for each comparison; effect size reflects the magnitude of differences found, whereas statistical significance examines whether the findings are likely to be due to chance. Another limitation relates to the inherent challenges associated with accurately modeling a biophysical system. In this study, IC and ISO parameters were obtained from the ball-&-sticks model, and a relatively new framework (COMMIT2) was used to assess structural connectivity. As the aim of this study was to assess changes in these metrics in MCI, no comparisons were performed with other advanced modeling methods. While each method has unique advantages and limitations, future work should compare various methods in the context of neurodegeneration, though validation will ultimately be critical for definitive interpretation of these findings.

In conclusion, this study shows significant differences in WM microstructural integrity between MCI and HC cohorts using three complementary methods: comparison of voxel-wise IC and ISO metrics, structural connectivity, and graph theory global metrics. At the local level, decreases in the intracellular compartment in MCI were observed across many white matter tracts, suggesting reduced white matter integrity, while increases in the isotropic compartment can be attributed to neurodegeneration and were also observed in MCI. At the global level, decreases in structural connectivity were observed in MCI in regions consistent with frontoparietal network dysfunction. Network-based metrics showed decreased global efficiency in MCI, demonstrating that changes occur over both local and global scales in MCI and may play a contributory role in cognitive impairment.

## Data Availability Statement

Publicly available datasets were analyzed in this study. This data can be found here: http://adni.loni.usc.edu/.

## Ethics Statement

The studies involving human participants were reviewed and approved by the Albany Medical Center Committee on Research Involving Human Subjects Institutional Review Board, Boston University Medical Campus and Boston Medical Center Institutional Review Board, Butler Hospital Institutional Review Board, Cleveland Clinic Institutional Review Board, Columbia University Medical Center Institutional Review Board, Duke University Health System Institutional Review Board, Emory Institutional Review Board, Georgetown University Institutional Review Board, Health Sciences Institutional Review Board, Houston Methodist Institutional Review Board, Howard University Office of Regulatory Research Compliance, Icahn School of Medicine at Mount Sinai Program for the Protection of Human Subjects, Indiana University Institutional Review Board, Institutional Review Board of Baylor College of Medicine, Jewish General Hospital Research Ethics Board, Johns Hopkins Medicine Institutional Review Board, Lifespan – Rhode Island Hospital Institutional Review Board, Mayo Clinic Institutional Review Board, Mount Sinai Medical Center Institutional Review Board, Nathan Kline Institute for Psychiatric Research & Rockland Psychiatric Center Institutional Review Board, New York University Langone Medical Center School of Medicine Institutional Review Board, Northwestern University Institutional Review Board, Oregon Health and Science University Institutional Review Board, Partners Human Research Committee Research Ethics, Board Sunnybrook Health Sciences Centre, Roper St. Francis Healthcare Institutional Review Board, Rush University Medical Center Institutional Review Board, St. Joseph’s Phoenix Institutional Review Board, Stanford Institutional Review Board, The Ohio State University Institutional Review Board, University Hospitals Cleveland Medical Center Institutional Review Board, University of Alabama Office of the IRB, University of British Columbia Research Ethics Board, University of California Davis Institutional Review Board Administration, University of California Los Angeles Office of the Human Research Protection Program, University of California San Diego Human Research Protections Program, University of California San Francisco Human Research Protection Program, University of Iowa Institutional Review Board, University of Kansas Medical Center Human Subjects Committee, University of Kentucky Medical Institutional Review Board, University of Michigan Medical School Institutional Review Board, University of Pennsylvania Institutional Review Board, University of Pittsburgh Institutional Review Board, University of Rochester Research Subjects Review Board, University of South Florida Institutional Review Board, University of Southern, California Institutional Review Board, UT Southwestern Institution Review Board, VA Long Beach Healthcare System Institutional Review Board, Vanderbilt University Medical Center Institutional Review Board, Wake Forest School of Medicine Institutional Review Board, Washington University School of Medicine Institutional Review Board, Western Institutional Review Board, Western University Health Sciences Research Ethics Board, and Yale University Institutional Review Board. The patients/participants provided their written informed consent to participate in this study.

## Author Contributions

MB downloaded the data for this study and takes responsibility for the integrity of the data and the accuracy of the data analysis, performed the statistical analysis, and wrote the manuscript. SS and AD developed the COMMIT2 algorithm and drafted the manuscript. RRW drafted the manuscript. AMS drafted the manuscript and supervised the whole study. All authors critically reviewed the article and approved the final manuscript.

## Conflict of Interest

The authors declare that the research was conducted in the absence of any commercial or financial relationships that could be construed as a potential conflict of interest.

## Publisher’s Note

All claims expressed in this article are solely those of the authors and do not necessarily represent those of their affiliated organizations, or those of the publisher, the editors and the reviewers. Any product that may be evaluated in this article, or claim that may be made by its manufacturer, is not guaranteed or endorsed by the publisher.

## Footnotes

1
http://adni.loni.usc.edu/

2
https://surfer.nmr.mgh.harvard.edu/

3
https://www.nitrc.org/projects/robex

4
https://www.mrtrix.org/

5
https://fsl.fmrib.ox.ac.uk/

6
http://stnava.github.io/ANTs/

7
http://www.R-project.org/
